# Identification of Ribonuclease 6 as an immunoinflammatory key gene associated with the glomerular injury in diabetic nephropathy

**DOI:** 10.1038/s41598-022-24289-0

**Published:** 2022-11-16

**Authors:** Tongyi Li, Yanmei Wang, Chan Zhu, Yunjiao Yang, Cong Long, Qiu Chen

**Affiliations:** Hospital of Chengdu University of Traditional Chinese Medicine, Chengdu, 610072 China

**Keywords:** Endocrine system and metabolic diseases, Nephrology

## Abstract

Diabetic nephropathy is one of the major causes of end-stage renal disease, and the pathogenesis of the disease has not been elucidated. While the immunoinflammatory response plays an essential role in the progression of diabetic nephropathy. Glomerular expression dataset in diabetic nephropathy was obtained from the GEO database. Differentially expressed genes were identified and functional enrichment analysis was performed to find genes associated with immunity and inflammation from them. The hub genes of immunoinflammatory were identified using MCODE after establishing the PPI network and gene expression was verified with diabetic nephropathy model rats. Xcell was used to assign immune cells to diabetic nephropathy glomerular samples to detect significant changes in immune cells and to analyze correlations with the hub gene. We found 120 DEGs associated with immunity and inflammation, Ribonuclease 6 was the Hub gene with the highest MCODE score. Xcell analysis revealed significant changes of immune cells in DN glomeruli, including upregulated Activated DCs, Conventional DCs, CD4+ Tem, Epithelial cells, Macrophages, Macrophages M1, and Memory B-cells. RNase6 expression showed the highest positive correlation with Macrophages M1, Activated DCs, and Conventional DCs. We verified through the Nephroseq v5 database that RNase6 expression was elevated in DN glomeruli and negatively correlated with glomerular filtration rate. Animal studies revealed that the kidney of DN model rats showed increased RNase6 expression together with inflammatory factor TNF-alpha and chemokine MCP-1. Our study identified RNase6 as a diagnostic and prognostic biomarker for diabetic nephropathy and found that it may play an essential role in the immunoinflammatory damage to the glomerulus.

## Introduction

Diabetic nephropathy (DN) is a common complication of diabetes and is considered a leading cause of chronic kidney disease (CKD) and end-stage renal disease (ESRD)^1^. In DN, hyperglycemia leads to glomerular damage, which in turn leads to renal injury through different mechanisms, such as mesangial proliferation, immune cell infiltration, and expression of inflammatory mediators^2^. Glomerular damage is one of the main pathological features of DN. There is considerable evidence that inflammatory responses and infiltrating immune cells play an important role in the pathogenesis of diabetic nephropathy and disease progression through the immune system^3^. In addition, the range of responses generated by inflammation plays a key role in the development and progression of diabetic nephropathy and the modulation of inflammation and associated immune activity is emerging as a therapeutic strategy^4,5^. The pathogenesis of DN is complex, and further elucidation of the pathological changes and mechanisms of DN and the search for new therapeutic targets can help improve clinical management.

Bioinformatics analysis can process large amounts of data in a short period and provide valuable information about a disease. In this study, a microarray dataset of diabetic nephropathy glomeruli was downloaded from the Gene Expression Omnibus (GEO) database and differentially expressed genes (DEGs) associated with inflammation and immunity were screened from it. We performed a cell type enrichment analysis of 64 immune and stromal cell types for microarray data using the Xcell online tool^6^ to investigate differences in immune cell infiltration between DN and healthy glomeruli. We also used online databases and animal trials to verify the clinical value of the hub gene and its relationship with inflammation and immunity.

## Materials and methods

### Microarray data processing

Transcriptome dataset GSE30528 was obtained from NCBI (National Center for Biotechnology Information) Gene Expression Omnibus (GEO, http://www.ncbi.nlm.nih.gov/geo/) database.GSE30528 (GPL571 Affymetrix Human Genome U133A 2.0 Array) consists of 9 DN Glomeruli samples and 13 control Glomeruli samples. The transcriptome dataset was processed using the GEO2R tool^7^ which is based on the “limma” R package. Probes that did not map to genes were removed. DEGs were identified based on adjusted P value < 0.01 and |log FC|> 1.

### Screening for target genes

Gene ontology enrichment analysis of the above DEGs was performed using the online tool Metascape^8^. First, we retrieved GO terms for immunity and inflammation from the Gene Ontology Resource (http://geneontology.org/) using the search terms "immune", "immunity", "inflammatory", and "inflammation", and the results are downloaded separately. Then we intersected the downloaded GOterms of interest with the GO enrichment results of Metascape, and the genes involved in these GOterms were considered to be target DEGs involved in immune or inflammatory processes. Importing the relevant genes into the STRING database (https://string-db.org/) and interaction score (medium confidence) > 0.4 was considered statistically significant. The PPI (protein–protein interaction) network of the genes in concern was generated and then visualized and analyzed with Cytoscape software (version 3.8.2, http://www.cytoscape.org/). The main functional modules and key biomarkers in the PPI are identified by the MCODE plugin. The HUB gene is selected from the module with the highest score.

### Enrichment and immune analysis of target genes

We used "clusterProfiler"^9^ and "ggplot2" R packages to enrichment analyze and visualize the GO function and KEGG pathway^10^ of target genes based on their LogFC values. We also used the ClueGO plug-in in Cytoscape to investigate the immune-related processes of the obtained inflammation-associated target genes.

### Immune and stromal cell analyses

Cell type enrichment scoring was performed with the gene expression dataset to predict the various cell types that may be involved in glomerular injury in diabetic kidneys. xCell^6^ used single sample gene set enrichment analysis (ssGSEA) to score cell types on enrichment analysis. The online platform can use gene expression data to calculate enrichment scores for 64 cell types, including 34 immune, 30 stromal and other cells, and to analyze intergroup differences. The scores obtained can be further analyzed and visualized.

### RNase6 verification and related analysis

Many immune cells promote inflammatory responses during glomerular injury in diabetic nephropathy and promote the production of multiple inflammatory factors^11^. Meanwhile, the alteration of PBMCs (peripheral blood mononuclear cells) can reflect the potential role of the immune system in diabetic nephropathy^12^. Transcriptome datasets from PBMCs of patients with diabetic kidney disease GSE142153 and transcriptome datasets of Human Diabetic Kidney Disease GSE30122 were used for receiver operating curve (ROC) analysis. ROC curves were plotted using the "pROC" package in R software, and the area under the curve > 0.7 was considered valuable. Then, we used the Woroniecka Diabetes Glom dataset in the Nephroseq v5 online database (http://v5.nephroseq.org) to validate the correlation between RNase6 (Ribonuclease 6) expression in the glomeruli and clinical traits of patients with diabetic nephropathy.

### Single cell RNA sequencing data analysis

Firstly, the Human Diabetic Nephropathy Single Cell Transcriptomic Landscape dataset released by Wilson et al.^13^ was selected to obtain the expression of the key gene RNase6 among different cell subpopulations in the control and DN groups using the Kidney Interactive Transcriptomics (http://humphreyslab.com/SingleCell/) online analysis platform. The dataset GSE195460 from the GEO database was then selected for analysis. The downloaded kidney snRNAseq samples from patients with diabetic nephropathy were processed using the "Seurat" package, and t-SNE was used to reduce the dimensionality for preliminary analysis of cell clustering. The expression of RNase6 and some immune cell marker genes in each cell subpopulation was then analyzed to predict their association, and the results were visualized using a bubble plot.

### Animal experiments

10 male SD rats (8–10 weeks old) were obtained from the Animal Experiment Center of West China Hospital, Sichuan University (Chengdu, China). 5 rats were randomly selected as the diabetic nephropathy model group, and then streptozotocin (STZ) 80 mg/kg was injected intraperitoneally to cause the disease. Blood was collected from the tail vein 48 h after STZ intraperitoneal injection, 24 h urine volume was recorded and 24 h urine protein was measured in rats after random blood glucose testing for 3 consecutive days were greater than 16.7 mmol/L. The DN model was successfully constructed when the urine volume was > 150% of the original urine volume and the urine protein volume was > 30 mg/24 h. After 8 weeks of DN rat model formation, rats are euthanized using intraperitoneal sodium pentobarbital. Animal studies were approved by the Experimental Animal Ethics Committee of West China Hospital of Sichuan University (Approval No: 20220205001). We confirm that the ARRIVE guidelines were complied with during the experiment. We confirm that all experiments were conducted under specified guidelines and regulations related to the ethics of animal experiments.

### Pathological histology and transmission electron microscope

Kidneys were fixed in 10% buffered formaldehyde, embedded in paraffin, and processed for sectioning. Pathological changes were detected by hematoxylin and eosin staining. Glomerular lesions and mesangial matrix expansion were evaluated with PAS (periodic acid/Schiff) staining to verify that the DN model in STZ-induced SD rats was successfully constructed. Image acquisition was performed using Pannoramic Digital Slide Scanners (3DHISTECH Ltd.). Prefixed with a 3% glutaraldehyde, then the tissue was postfixed in 1% osmium tetroxide, dehydrated in series acetone, infiltrated in Epox 812 for a longer, and embedded. The semithin sections were stained with methylene blue and the ultrathin sections were cut with diamond knife, and stained with uranyl acetate and lead citrate. Sections were examined for microscopic glomerular lesions with JEM-1400-FLASH transmission electron microscope.

### RNA extraction and real-time PCR analysis

Total rat kidney RNA was extracted with RNA Trizol Reagent (Bomei, BM1144) and other reagents, and reverse transcribed into cDNA with PrimeScript RT reagent Kit (Takara Bio Inc., RR047A). Real-time polymerase chain reaction (RT-PCR) was performed using a QuantStudio TM3 instrument (Thermo Fisher Scientific) and CT (Threshold cycle) values of the PCR process were analyzed using Thermo Scientific PikoReal software. Comparative gene expression was calculated using the Livak method in this experiment. The following primers were used: MCP-1 (Monocyte Chemoattractant Protein-1) forward 5′-ctcacctgctgctactcattcactg-3′, reverse 5′-cttctttgggacacctgctgctg-3′; RNase6 forward 5′-agccatgcgtggtgtcaacaac-3′, reverse 5′-ggcagttcttccgaccgttcttg-3′; TNF-alpha (Tumor Necrosis Factor-Alpha) forward 5′-atgggctccctctcatcagttcc-3′, reverse 5′-cctccgcttggtggtttgctac-3′.

### Statistical analyses

R Studio and GraphPad Prism 9 are used for graphing and statistical analysis. Student’s t-test (unpaired, two-tailed) was employed for comparisons between the two groups. The P value < 0.05 was considered statistically significant (*P < 0.05, **P < 0.01, ***P < 0.001).

## Results

### Identification of DEGs in DN glomerulopathy

**Figure 1:**
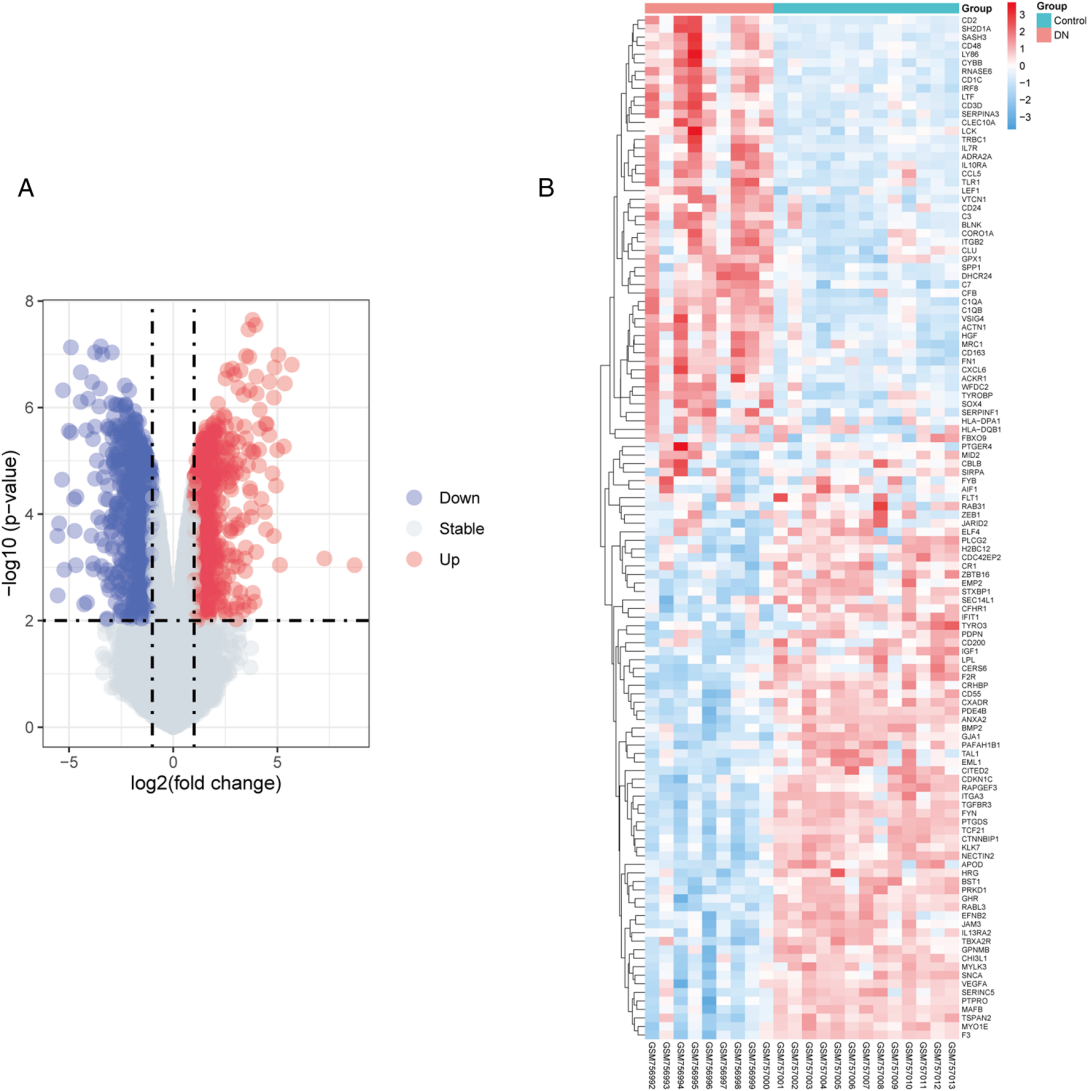
Comparison of differentially expressed genes between the control and DN groups. (**A**) Volcano plot of all differentially expressed genes for dataset GSE30528. (**B**) Heatmap of 120 genes associated with immunity and inflammation.

After analysis and processing of the microarray dataset GSE30528, a total of 517 DEGs were identified and screened, including 108 up-regulated genes and 409 down-regulated genes (Fig. 1A).

### Identification of Hub Genes

**Figure 2:**
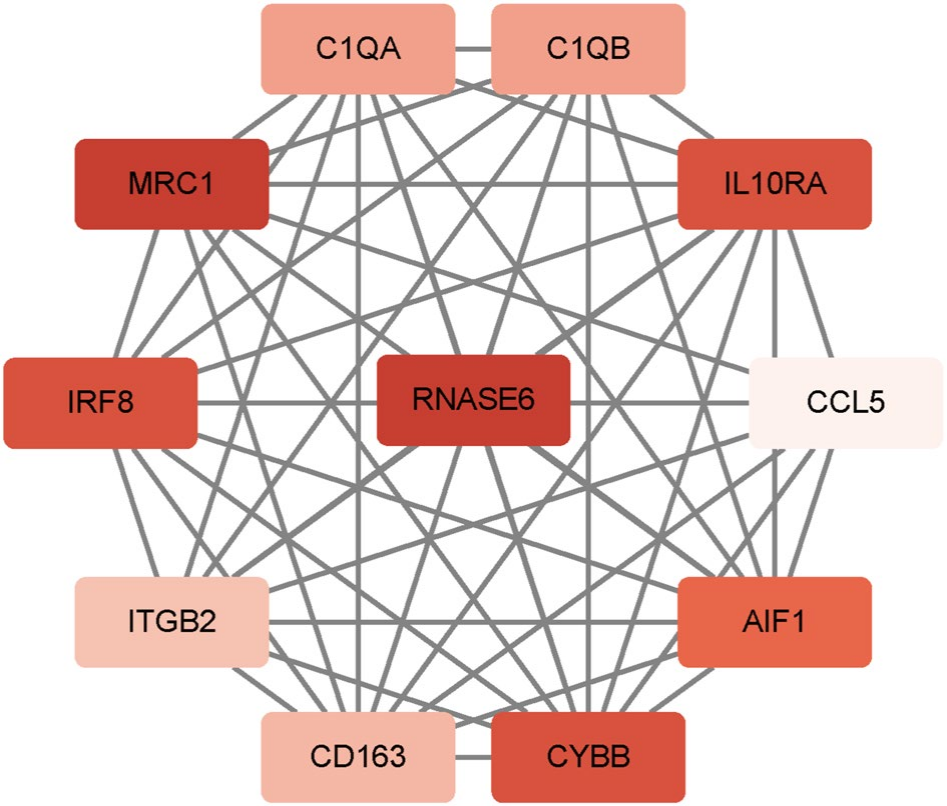
The key functional module with the highest MCODE score.

We collected 120 genes in GO terms regarding immune and inflammation after analysis (Fig. 1B). The PPI network generated after the import of genes into STRING is visualized and analyzed by Cytoscape software. The MCODE plugin is used to identify dominant modules. The MCODE plug-in was used to qualify the main functional modules and obtained one cluster with a score > 9 that was considered to be key functional module (Fig. 2). In this cluster, RNase6 was identified as the seed node with the highest Mcode Score, which is also the hub gene (Table 1).

**Table 1:** Genes in the main functional module identified by MCODE.

Symbol	MCODE:Node Status	LogFC
CYBB	Clustered	1.0823763
AIF1	Clustered	− 1.6724402
MRC1	Clustered	1.4041473
ITGB2	Clustered	1.2886009
C1QA	Clustered	1.9222762
CD163	Clustered	1.9733481
CCL5	Clustered	1.7204926
C1QB	Clustered	2.2046858
RNASE6	Seed	1.4200724
IRF8	Clustered	1.5832472
IL10RA	Clustered	1.4681625

### Functional analysis of related genes

**Figure 3:**
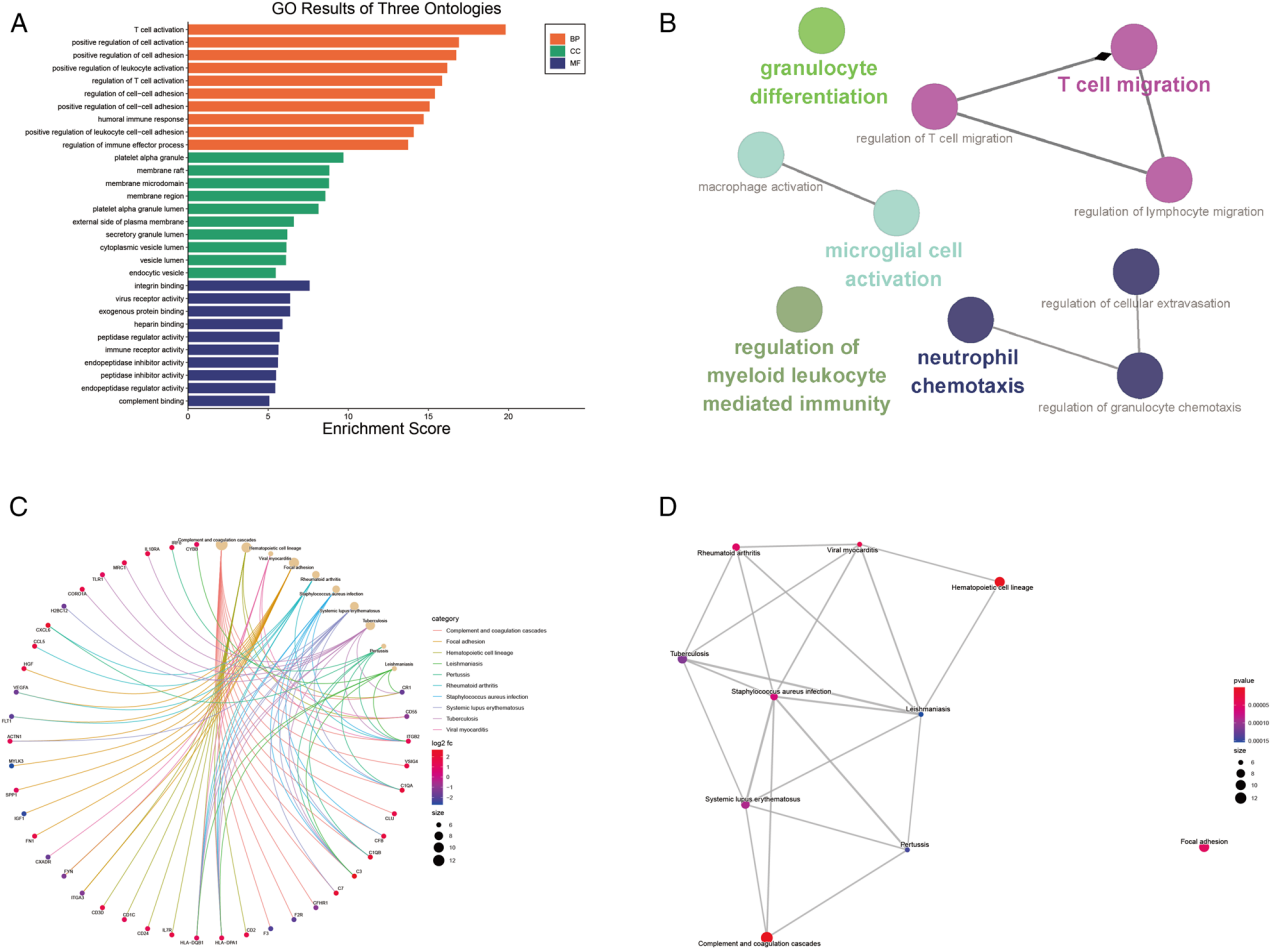
Functional enrichment analyses. (**A**) GO term enrichment analysis results for the top 30 GO terms, including the top 10 BPs, CCs, and MFs. (**B**) Enrichment analysis of immune processes for genes associated with inflammation. (**C**,**D**) KEGG^10^ pathway enrichment results and their interrelationships.

GO analysis indicated that the related genes were significantly enriched in biological processes (BP), including T cell activation, positive regulation of cell activation, and humoral immune response. Additionally, KEGG analyses revealed that the genes were mainly enriched in complement and coagulation cascades, indicating that it may play an important role in glomerular injury caused by immune inflammation in diabetic nephropathy (Fig. 3). The ClueGO plug-in of Cytoscape was used to study and visualize the genes in GO terms related to inflammation to further analyze the immune processes involved in inflammation (Fig. 3).

### Immune and stromal cell analyses

**Figure 4:**
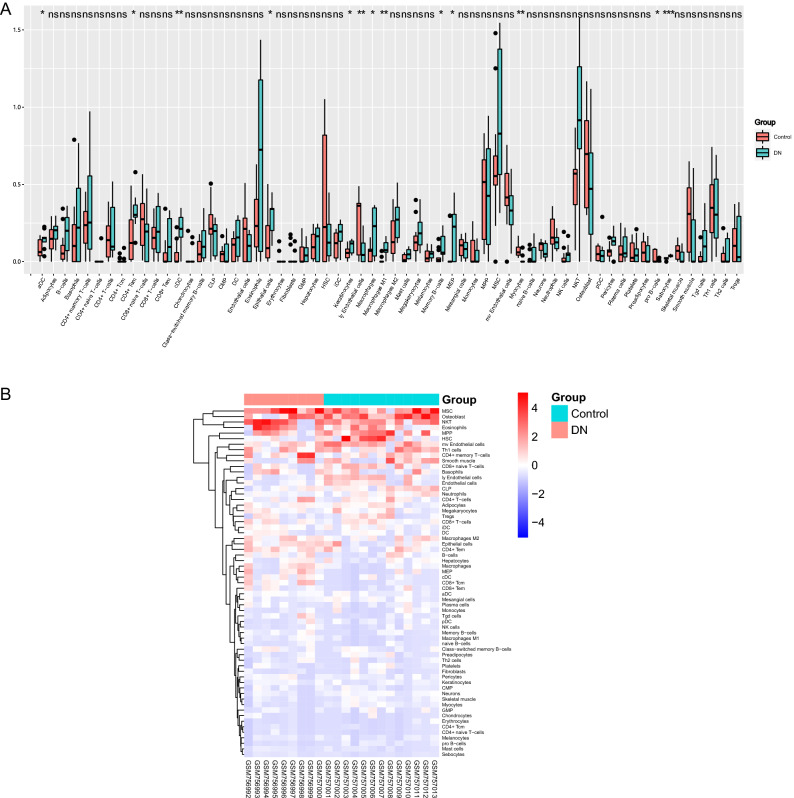
Immune cell infiltration in DN glomeruli analyzed by xCell. (**A**) Comparison of xCell scores of 64 cell types in the glomeruli between DN and control group in the GSE30528 dataset. (**B**) The heatmap represents the cell type enrichment score for each cell type of all samples.

We used xCell to generate cell type enrichment scores from gene expression data to identify cell types that may be involved in the glomerular injury of diabetic nephropathy (Fig. 4). The 64 cell types include 34 immune cells and 30 other cells such as stromal cells. Box plots of cell infiltration showed higher levels in the infiltration of activated DCs, conventional DCs, CD4+ Tem (effective memory T Cell), epithelial cells, keratinocytes, macrophages, macrophages M1, memory B-cells, and MEP (megakaryocyte–erythrocyte progenitors) in the glomeruli of diabetic nephropathy than in the healthy controls (P < 0.05). Among the immune cells, the infiltration level of pro B-cells cells was lower in the diabetic nephropathy group than in the control group (P < 0.05). The enrichment fractions of most cells were not significantly different between the two groups except for B-cell subpopulations, DC subpopulations, and macrophage subpopulations.

### Correlation between RNase6 expression and infiltrating immune cells

**Figure 5:**
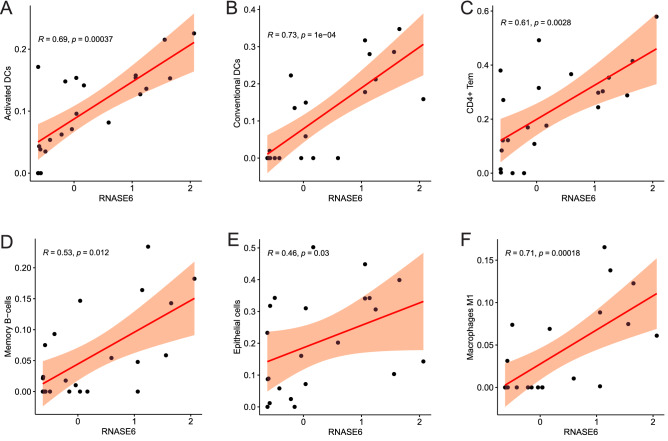
Correlation of the key gene with differentially expressed immune cells.

Among the key genes identified, RNase6, the gene with the highest MCODE score, was identified as a hub gene associated with immunity and inflammation. Correlation analysis showed that RNase6 expression was correlated with Activated DCs (R = 0.69, P = 0.00037), conventional DCs (R = 0.73, P = 1e−04), CD4+ Tem (R = 0.61, P = 0.0028), memory B-cells (R = 0.53, P = 0.012), epithelial cells (R = 0.46, P = 0.03), and macrophages M1 (R = 0.71, P = 0.00018) cells showed a positive correlation in the level of infiltration (Fig. 5).

### Single cell RNA sequencing data analysis

**Figure 6:**
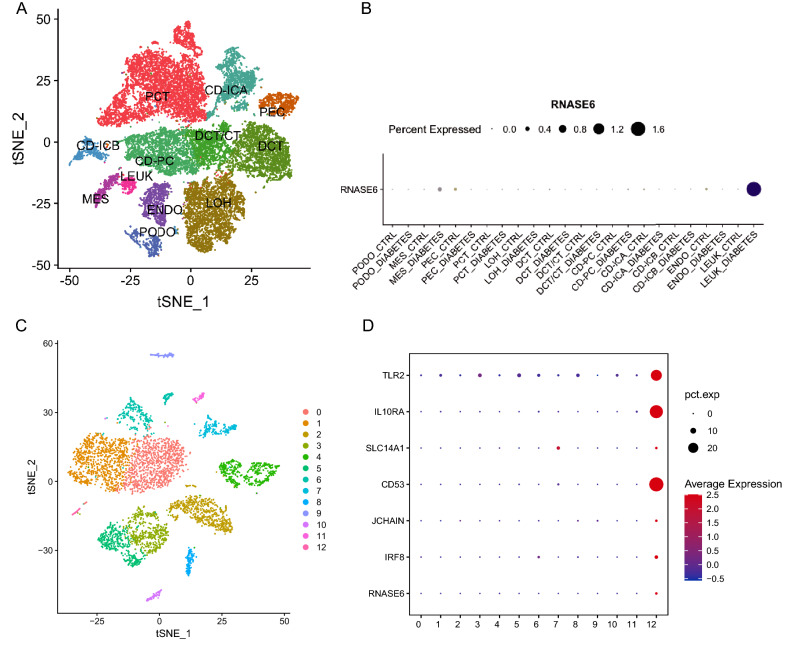
(**A**) t-SNE dimensionality reduction analysis and information on cell subgroups. (**B**) Expression of RNase6 among different cell subpopulations in the two groups. (**C**) t-SNE dimensionality reduction analysis of Single cell sequencing samples of diabetic nephropathy. (**D**) The expression of RNase6 and different immune cell markers in different cell subgroups.

Based on the results of cell subgroup labeling and analysis, it was shown that RNase6 was expressed higher in glomerular MES (mesangial cells) and LEUK (leukocytes) mainly in the diabetic group (Fig. 6A,B). single cell sequencing samples of diabetic nephropathy in dataset GSE195460 using t-SNE dimensionality reduction analysis revealed that the cells were divided into 13 subpopulations (Fig. 6C). RNase6 was mainly highly expressed in cluster 12, while some cell markers, such as M1 macrophage marker TLR2, dendritic cell markers JCHAIN and IRF8, monocyte markers CD53 and IL10RA, and renal epithelial cell marker SLC14A1 were also highly expressed in cluster 12 (Fig. 6D).

### Correlation between RNase6 expression and the clinical value of RNase6 in DN

**Figure 7:**
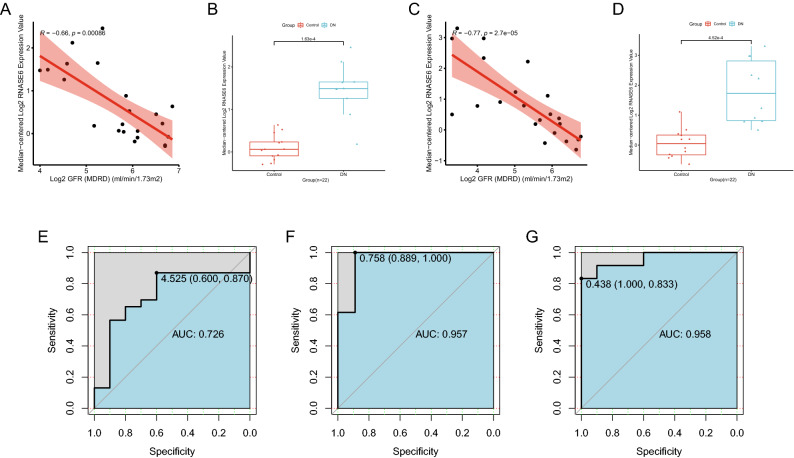
Correlation of RNase6 expression with glomerular filtration rate (GFR) in diabetic nephropathy and its diagnostic and prognostic value. (**A**) Correlation between RNase6 expression and GFR (Glomerulus). (**B**) Differences in RNase6 expression in the glomeruli of the control and DN groups. (**C**) Correlation between RNase6 expression and GFR (tubulointerstitium). (**D**) Differences in RNase6 expression in the tubulointerstitium between the control and DN groups. (**E**) ROC curve of RNase6 expression in DN (GSE142153,Peripheral blood mononuclear cell sample). (**F**) ROC curve of RNase6 expression in DN (GSE30528,Glomerulus). (**G**) ROC curve of RNase6 expression in DN (GSE30529,tubulointerstitium).

**Figure 8:**
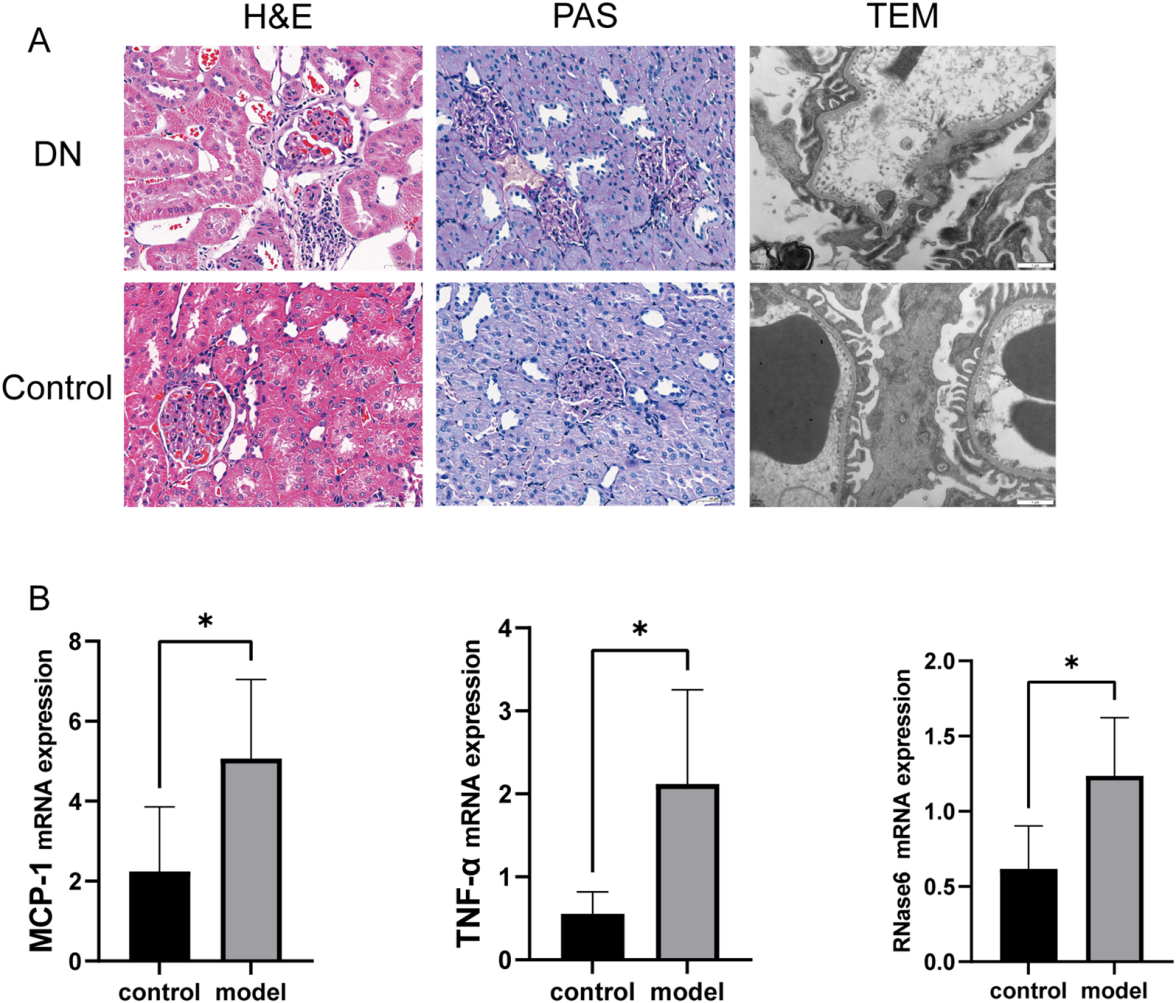
(**A**) Representative photomicrographs of TEM (magnification, ×20,000) and histology of renal sections stained with H&E, PAS (magnification, ×400) in the two groups. (**B**) The mRNA levels of RNase6, MCP-1, and TNF-alpha in rat kidney tissues were measured by RT-PCR (mean ± s.d., n = 10, *P < 0.05).

ROC analysis of RNase6 was performed using glomerular and tubulointerstitial sample expression data from the Nephroseq v5 online database and the dataset GSE142153 to determine the value of the gene. The results showed that the AUC values in the ROC analysis were all > 0.7, and RNase6 expression levels were significantly higher in the DN tubulointerstitium and glomerular. Meanwhile, RNase6 expression was negatively correlated with glomerular filtration rate (GFR) (R = 0.66, P = 0.00086). The results are represented in Fig. 7. PAS and HE staining of rat kidneys confirmed glomerular basement membrane thickening, basement membranes of the Bowman’s capsule thickening, lymphocytic infiltration, etc., proving that the experiment successfully caused diabetic nephropathy in rats (Fig. 8). The mRNA expression of TNF-alpha, MCP-1, and RNase6 in the DN group was significantly higher than that in the control group measured by RT-PCR (Fig. 8).

## Discussion

Diabetic nephropathy is a chronic disease that can lead to proteinuria, kidney failure, histopathy, and more. Also immune cells and chronic inflammation play an important role in the pathogenesis^5,14^. Diabetic nephropathy is associated with both systemic and localized inflammation of the kidney. In the course of renal injury in early diabetic nephropathy, it has been shown that proteinuria is associated with C-reactive protein (CRP)^15^, a marker of systemic inflammatory response, and MCP-1, a marker of renal inflammation, and that urinary MCP-1 levels can be an independent predictor of nephropathy progression^16^. Several molecules released by cellular damage due to metabolic impairment and altered hemodynamics in diabetic nephropathy are involved in both immune response and inflammatory response through different mechanisms^17^. Therefore integrating the immune response and the inflammatory response view for study may help us to further understand the mechanisms of the disease.

In this study, RNase6 was identified as an essential biomarker that has clinical value and can influence the immune and inflammatory response to DN. Based on the analysis of single-cell sequencing data we can speculate that RNase6 is involved in the process of glomerular immunoinflammatory damage in diabetic nephropathy and that its role in the mononuclear phagocytic system should be equally valued. RNase6 and M1 macrophages, dendritic cells, monocytes, renal epithelial cells, and glomerular mesangial cells are associated to some extent in diabetic nephropathy. This result also supports the conclusions we obtained from transcriptomic data and immune cell analysis from another perspective. Renal leukocyte infiltration is one of the features of diabetic nephropathy, and numerous immune cells belonging to leukocyte subpopulations such as macrophages and T lymphocytes are involved in the pathological process of diabetic nephropathy^17,18^. Human Ribonuclease 6 is a secreted protein belonging to the ribonuclease A (RNaseA) superfamily. Tissue distribution analysis showed that RNase6 was abundantly expressed in monocytes and neutrophils and that bacterial infection could induce the expression of this protein in vivo and exhibited antibacterial activity^19,20^. Also, macrophage M1 can express high levels of pro-inflammatory cytokines to enhance the clearance of pathogens^21^. The RNases family has been reported to have innate immune properties, and vertebrate RNases have evolved as a host defense family^22^. Current studies suggest that some members of the ribonuclease family have a clear defense immune role in the urinary system^23,24^, but there are fewer studies in primary endocrine system diseases and their complications.

The comprehensive assessment of immune cell infiltration in the glomeruli of DN patients showed that immune cells involved in both active and passive immunity are engaged in DN glomerular injury. Immune infiltration analysis showed that the expression of dendritic cells and macrophages in the glomeruli of diabetic nephropathy was significantly higher than that of controls. Thus, it is suggested that the renal mononuclear phagocytic system plays pivotal roles in pro-inflammatory and immune responses in this disease process^25,26^. Dendritic Cells (DCs) are the main antigen-presenting cells of bone marrow origin and play an immune role together with T cells. It has been shown that a high glucose environment can lead to the activation and maturation of dendritic cells^27^. However, all DC subgroups do not cause the same pattern of pathological damage to the kidney, varying according to the type and stage of disease development and the activation of different subgroups of DCs. Transplantation of MSCs (mesenchymal stromal cells) into rats with diabetic nephropathy significantly reduced the levels of CD103+CDC1s, while decreasing the expression of inflammatory factors TNF-alpha and MCP-1, which in turn attenuated renal injury through the inactivation of CD8+ T cells^3^. Kidney-infiltrating CD4+ T cells can stimulate macrophages by producing cytokines such as TNF-alpha. Moreover, CD4+ T cells can stimulate B cells to produce antibodies against circulating antigens that trigger complement cascade reactions in glomerular capillaries, causing immune and inflammatory injury^28^, which is also consistent with the results of our KEGG enrichment analysis. In terms of the complement system, activation of the lectin pathway in response to cell surface glycated proteins and hyperglycemia-induced glycosylation of complement regulatory proteins are both involved in the course of DKD, and both are associated with innate immunity^29^. Furthermore, macrophages can produce complement system factors causing kidney damage. Macrophages in glomeruli are commonly infiltrated in the early stages of DN and macrophages are pathogenic for the disease and can be mediated by a variety of chemokines, such as CCL2 (MCP-1)^30,31^. The level of chemokine MCP-1 in the urine gradually increases during diabetic nephropathy as macrophage infiltration and renal function decline, and its expression in renal tissue is associated with disease progression^32,33^. During diabetic nephropathy, infiltrating macrophages can polarize to a pro-inflammatory M1 phenotype and promote TNF-alpha expression, facilitating the inflammatory response of DN and leading to the destruction of the glomerular basement membrane and Bowman’s capsule^34,35^. The polarized typing of macrophages and the different inflammatory states of the organism can be reflected by the detection of peripheral blood mononuclear cell biomarkers^36^. In an animal model of diabetic nephropathy, T cells were found to release TNF-alpha and interferon to activate endothelial cells and macrophages to exacerbate the inflammatory response^37^. The role of B cells and related antibodies in glomerular damage is more well-known than that of T cells.

The expression of RNase6 was significantly and positively correlated with macrophage M1 and dendritic cells. The kidney contains a complex network of mononuclear phagocytes, including dendritic cells and macrophages, which play a key role in immune inflammation-mediated tissue injury and repair. Therefore, we speculate that RNase6 may promote the immune inflammatory response to glomerular injury through renal mononuclear phagocytic system. Our animal experiments showed that the expression of RNase6, TNF-alpha, and MCP-1 was elevated in the DN group, which provided preliminary evidence for our hypothesis.

Bioinformatics approaches were used to analyze the relationship of key genes with immune cells and inflammation, revealing that specific immune inflammatory responses play an essential role in the pathology of diabetic nephropathy. RNase6 was identified as a novel biomarker for diabetic nephropathy, and detection of it may reflect glomerular immune inflammatory damage in DN, which may have guiding significance for future studies. Furthermore, RNase6 expression was negatively correlated with GFR, which is a prognostic and diagnostic indicator of diabetic nephropathy. Targeted RNase6 immunotherapy may help improve the prognosis of DN patients. Nevertheless, complete trials and more samples are needed to elucidate the mechanisms underlying the action of RNase6 in diabetic nephropathy.

## Conclusion

Using bioinformatics analysis, we predicted glomerular immune cell infiltration in DN. RNase6 was identified as the hub gene, closely associated with immune inflammatory damage in diabetic nephropathy. In addition, RNase6 expression was most positively correlated with macrophages M1 and conventional DCs, activated DCs, thus we speculate that it plays an essential role in the exacerbation of glomerular injury of diabetic nephropathy by the renal mononuclear phagocytic system. RNase6 has been identified as a key biomarker for the diagnosis and prognosis of diabetic nephropathy, providing a potential target and novel evidence for further research and treatment of diabetic nephropathy.

## Data Availability

The datasets generated and analysed during the current study are available from the corresponding author on reasonable request.
